# Office design and occupational health – has research been left behind?

**DOI:** 10.5271/sjweh.4073

**Published:** 2022-12-30

**Authors:** Annu Haapakangas, David M Hallman, Eva L Bergsten

**Affiliations:** 1 Finnish Institute of Occupational Health, Helsinki, Finland; 2 University of Gävle, Gävle, Sweden; 3 Karolinska Institutet, Stockholm, Sweden

Tens of millions of workers in Europe work in office environments. Such a physical environment might seem fairly harmless compared to the exposures of many other work environments. Yet, the office design is associated with important occupational health-related outcomes, as demonstrated by increased sickness absence (1) and the risk of disability retirement (2) in traditional open-plan offices. Unfortunately, modern office designs, such as activity-based offices, have received limited attention in the field of occupational and public health. Even in traditional offices, the mechanisms behind the associations with sickness absence and work ability are poorly understood and research on particular health outcomes, such as mental health and musculoskeletal disorders, is very limited. Research continues to be fixated on the simplistic dichotomy of “open-plan” versus individual cellular offices, while the evolution towards more sophisticated and complex office designs has been under way for several decades. As the development of office design appears to have been be accelerated by the COVID-19 pandemic (3), research on the health implications of modern offices is severely lagging behind.

## Contemporary office design

For a few decades, the trend has been towards more space-efficient office designs that are based on desk-sharing instead of assigned rooms and workstations. This development is supported by many forces in society, including digitalization, increasingly flexible work arrangements, an emphasis on collaboration – and collaborative workspaces – as a key productivity factor in knowledge work, and growing attention on the environmental impacts of buildings. An activity-based office design is currently a common solution to address these different trends (4). Thus, workers are expected to switch between workspaces that are designed for different activities (eg, collaborative or concentrative tasks) depending on what they are doing. Before the pandemic, a typical activity-based office had desks for about 70% of employees (5, 6), but much lower targets of desks-to-employees ratios can be expected as a response to increased teleworking. Many organizations are also re-thinking the role of the office as teleworking appears to have permanently increased (3). Thus, it may not be just the amount of space, but also the types of activities that are supposed to take place at the office, that could change in the coming years.

Modern office designs are commonly confused with traditional open-plan offices. However, the dimensions of the office design that could be relevant to how workers experience and are affected by the environment are more complex. An activity-based office can be defined by (i) desk sharing (ie, lack of assigned spaces), (ii) the provision and flexible use of task-related workspaces, and (iii) the openness of the main work environment (6) although, in practice, there is plenty of variation in how the concept is applied and described. The location of the office is yet another dimension (7). While working from home has become a major topic of research during the COVID-19 pandemic (8–10), increased teleworking might also lead to new applications of coworking office designs (eg, near residential areas). Such offices often apply the activity-based design but are intended for the shared use of small businesses or individual workers from different organizations.

Different combinations of these dimensions can create physically and socially different work conditions in terms of both job demands (eg, distractions) and job resources (eg, social relations). For example, Gerdenitsch et al (11) found that environmental distractions decreased and interaction across teams increased after workers moved from shared office rooms to an activity-based office. The reference office type, however, is important as the risks and benefits of modern office designs appear different depending on which office type they are compared to (6, 12). For example, a controlled natural intervention study by Haapakangas et al (13) found increased job demands and decreased social support after a relocation from individual cellular offices to an activity-based office, while no effect was found for those who moved from an open-plan office to an activity-based office.

The desk-sharing feature appears to be important for more positive perceptions of the office environment in shared workspaces (14), possibly due to facilitating an active choice of suitable workspace. Such behaviour could potentially contribute to a higher fit between the person and the environment (11, 15) and higher perceived autonomy (16) as well as more physical activity in sedentary jobs (17–19). On the other hand, qualitative observations suggest that constantly switching workspace – with all related activities from planning the workday to setting up and clearing workstations – is an additional job demand (14, 20).

## Gaps in existing research

It is striking how little is known about the health implications of modern office designs. The research field is developing and particularly the number of high-quality studies is still small. For example, a systematic review on health, work performance and perceived work environment in activity-based offices that was published in 2019 (12) identified only 17 studies, many of which reported only descriptive information, were congress papers, or investigated only activity-based offices without any reference condition. Few of the included studies actually addressed health, indicating that perceived satisfaction with different aspects of the work environment and self-rated productivity have been the dominating outcomes of interest so far.

While research on office design has increased in recent years, studies on outcomes such as musculoskeletal disorders, sedentary behavior, mental health, and sickness absence are still rare and the evidence is equivocal. Studies often lack in the definition and description of the investigated office and pay insufficient attention to confounding factors. The use of registry data and other objective measures has been rare. Longitudinal studies on the effects of office redesign tend to lack control groups and rarely exceed one-year follow-ups. The individual-level variation in the perceptions and effects of modern offices has become a topic of research in recent years (11, 15, 21), but there is still little, if any, knowledge on vulnerable groups or individual risk factors for adverse effects on health. Even though several systematic reviews have addressed the effects of office design (1, 12, 17, 22, 23), they have not critically evaluated the methodological deficiencies of the field and their implications on the interpretation of the existing evidence. The evidence pointing towards impaired well-being and health in traditional open-plan offices cannot be directly generalized to modern office designs, such as activity-based offices. Given the limited and partly inconsistent research, the relations between modern office designs and health are still largely unknown.

## Suggestions for future research

The limitations of the existing knowledge are partly related to the interdisciplinary nature of this research topic. Producing information that is both relevant and reliable requires combining building sciences (eg, facility management, architecture) with expertise from behavioral, occupational and health sciences.

Thus, more research with a strong occupational health perspective is crucial to raise the quality of scientific knowledge in this area of research. Questions on the physical work environment should be included in large epidemiological studies of occupational health and well-being. Research is needed on specific health outcomes (eg, mental and musculoskeletal health), as well as antecedents (eg, health behaviors) and consequences of poor health (eg, sickness absence, productivity loss). Studies should also pay attention to the explaining mechanisms and the determinants of person-environment fit in different worker groups and jobs, including the identification of potentially vulnerable groups. In particular, the physical office environment needs to be included in research on hybrid work as it is a part of the same phenomena.

Secondly, strong interdisciplinary collaboration is necessary. A careful definition of the investigated workspaces, including on-site observations and specific parameters of office design and use when possible (eg, space-efficiency ratios, data from occupancy sensors), is important to accurately measure the physical environment. In our view, generic terms, such as “open-space office” or “open-plan design” should be avoided because they blur differences between traditional and modern office concepts and mask other potentially important features. Studies that define offices in terms of several features will also more likely be relevant to evaluating future office types (which are not yet known). Survey- and registry-based studies need to be complemented with objective measurements of office design parameters and physical indoor environment. For example, perceived office noise is associated with sickness absence (24), but noise disturbance depends considerably on the quality of the room acoustic design, not only the office type (25).

Finally, the gaps in knowledge suggest that work environmental regulation and occupational safety and health policies may not be up to date in terms of relevant risk factors in modern office work. Public authorities, such as the European Agency for Safety and Health at Work (EU-OSHA) are drawing attention to the rapid changes in the working life, such as the impact of digitalization on work and workplaces and the associated occupational safety and health challenges and opportunities. If teleworking is becoming the “new normal” and offices are re-designed in response, there is an urgent need for evidence-based guidelines and tools on how to meet employers’ responsibilities to prevent work-related disorders and promote good health and well-being at work. Interestingly, health-promoting workspaces are already becoming a selling point for consultants and workplace designers in the post-pandemic working life. But if research is (still) left behind, organizations could be basing very expensive decisions on assumptions rather than evidence.
